# Attempted Arm and Hand Movements can be Decoded from Low-Frequency EEG from Persons with Spinal Cord Injury

**DOI:** 10.1038/s41598-019-43594-9

**Published:** 2019-05-09

**Authors:** Patrick Ofner, Andreas Schwarz, Joana Pereira, Daniela Wyss, Renate Wildburger, Gernot R. Müller-Putz

**Affiliations:** 1 Graz University of Technology, Institute of Neural Engineering, BCI-Lab, Graz, Austria; 2 AUVA rehabilitation clinic, Tobelbad, Austria

**Keywords:** Spinal cord injury, Biomedical engineering, Motor cortex, Brain-machine interface

## Abstract

We show that persons with spinal cord injury (SCI) retain decodable neural correlates of attempted arm and hand movements. We investigated hand open, palmar grasp, lateral grasp, pronation, and supination in 10 persons with cervical SCI. Discriminative movement information was provided by the time-domain of low-frequency electroencephalography (EEG) signals. Based on these signals, we obtained a maximum average classification accuracy of 45% (chance level was 20%) with respect to the five investigated classes. Pattern analysis indicates central motor areas as the origin of the discriminative signals. Furthermore, we introduce a proof-of-concept to classify movement attempts online in a closed loop, and tested it on a person with cervical SCI. We achieved here a modest classification performance of 68.4% with respect to palmar grasp vs hand open (chance level 50%).

## Introduction

Persons with cervical spinal cord injury (SCI) have lost the majority of voluntary motor control functions. In addition to paralysis of the lower limbs, upper limb functionality is usually severely limited. Brain-computer interfaces (BCIs)^1^ in combination with upper-limb motor neuroprostheses^2,3^ have been proposed as a remedy^4–6^. A BCI can detect user induced changes in brain-signals and transform them into control signals for neuroprostheses or robotic arms^5–16^. Brain signals can be recorded invasively or non-invasively for this purpose. In the present work, we focus on and apply the non-invasive electroencephalography (EEG) recording technique. EEG-based BCIs for neuroprostheses control rely typically on changes of oscillations originating from sensorimotor areas^17,18^ to detect and differentiate executed, imagined or attempted movements involving different body parts^15^. However, oscillation-based BCIs could impose non-intuitive control paradigms (e.g. repetitive foot motor imagery to control a hand function). In recent years, low-frequency time-domain signals have also gained attention in the BCI field as these have been shown to encode even more information about movements such as trajectories^19–23^, see^24^ for a review. Movement-related cortical potentials (MRCPs)^25,26^ in particular were shown to encode, e.g., reaching directions/targets^27–29^, or force^30^.

MRCPs are particularly interesting when designing an intuitive control paradigm for a BCI. For example, MRCPs encode even various single movements of the same limb, such as hand open, hand close, or different grasp types^31–33^, which could be detected by an EEG-based BCI and transformed into movements via a neuroprosthesis. As a result of this a person with SCI would only need to attempt to open the right hand in order to actually open it, and analogously, to close it. This eventually allows a natural control paradigm. In this work, we thus analyzed MRCPs in participants with SCI during attempted movements.

This work comprises two parts. In the first part, we analyzed whether attempted arm and hand movements of participants with SCI can be classified from MRCPs. We have shown in our previous work^31,32^ that low-frequency time-domain EEG signals (which capture MRCPs) encode information about hand and arm movements of the same limb in able-bodied participants. The translation of these results to participants with SCI, however, is still lacking. MRCPs in participants with SCI are present during movement imagination and movement attempts but there is evidence that they are altered^34–36^. As a result of this they may not be encoding the same information as in able-bodied participants. We therefore analyzed whether single movement attempts (i.e. hand open, palmar grasp, lateral grasp, supination, and pronation) can be classified based on low-frequency time-domain EEG signals in participants with SCI. Furthermore, we analyzed movement-related differences of the neural correlate.

In the second part, we propose a proof-of-concept of an MRCPs-based online classifier for self-paced attempted hand movements (hand open vs palmar grasp). In this proof-of-concept study, we describe the necessary adaptations of the training paradigm and the classifier, and present the online results of two sessions in a participant with SCI.

## Results

### Movement classification

#### Classification accuracies

**Figure 1:**
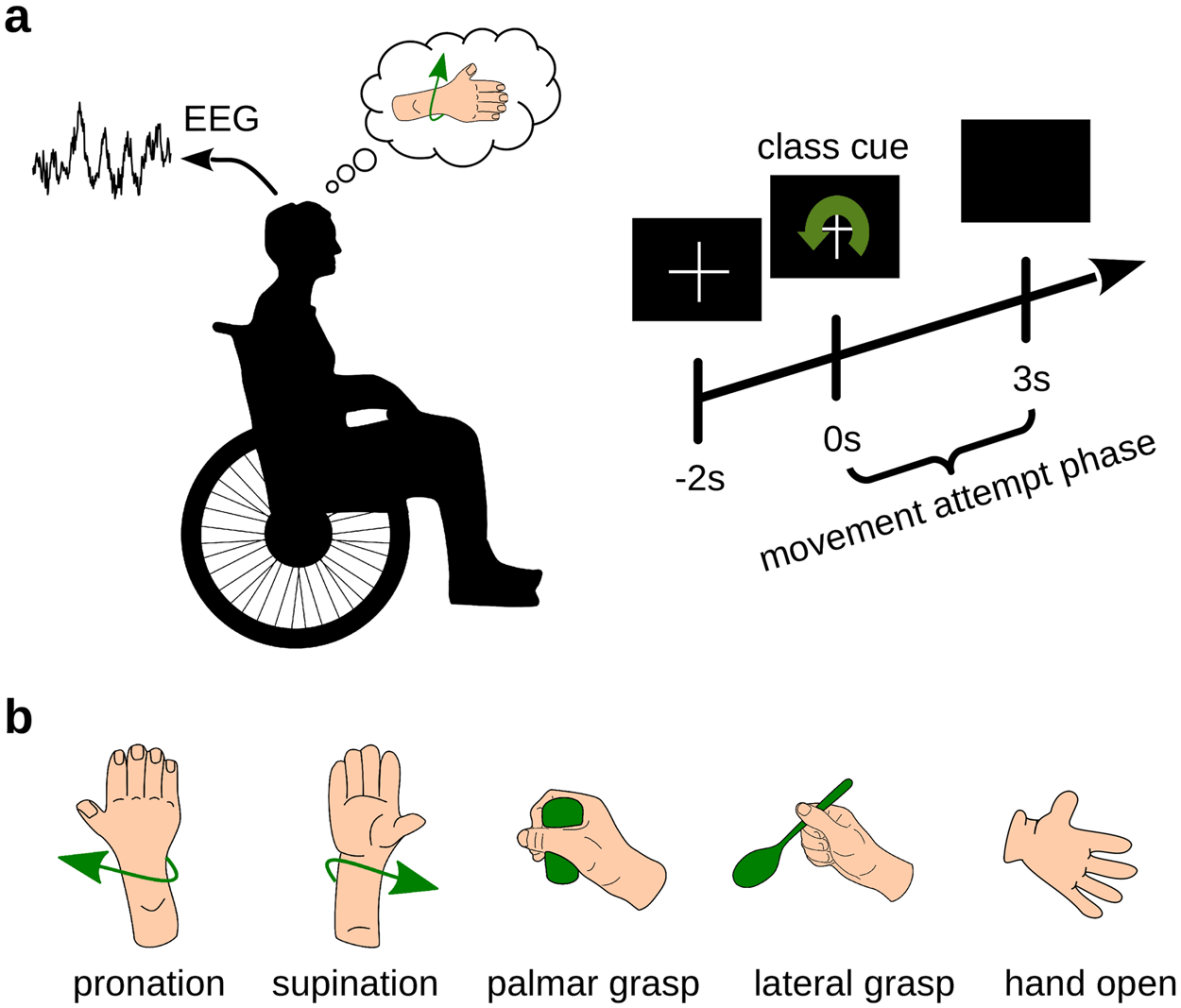
Offline Paradigm. (**a**) Participants with SCI sat in their wheelchairs and attempted the movement requested on a computer screen. (**b**) Illustration of the attempted movements.

**Figure 2:**
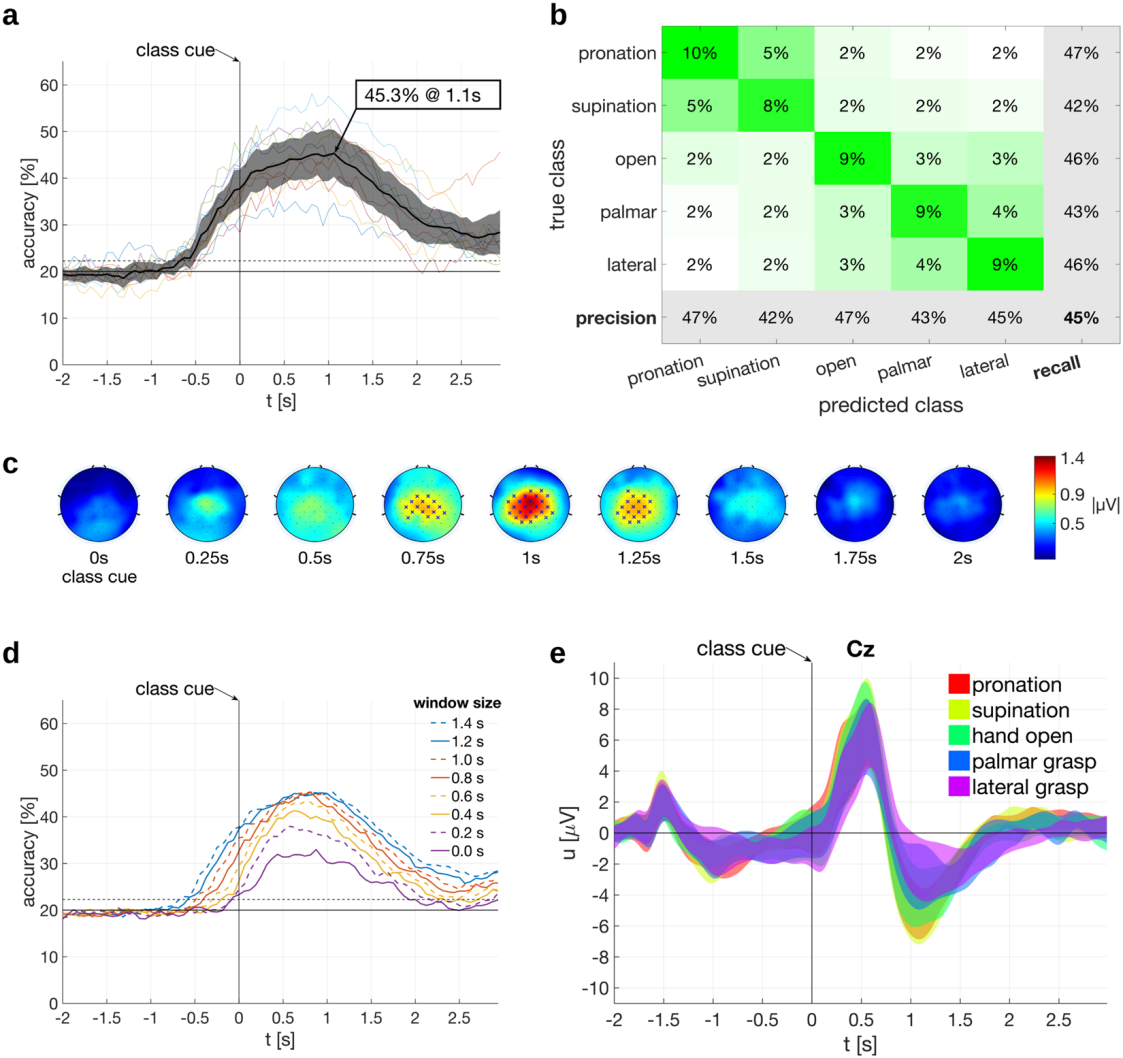
Offline Results for the full set of participants (n = 10). All times are relative to the trial start (**a**) Classification accuracy as a function of time. Grand-average classification accuracy and its respective 95% confidence interval is shown in black, individual classification accuracies are shown in thin, colored traces (feature extraction window size = 1.4 s). The grand average classification accuracy peak is marked. (**b**) Confusion matrix at peak grand average classification accuracy. (**c**) Difference Topoplots. Statistically significant differences on electrodes are marked with an “x”. (**d**) Grand average classification accuracies for different feature extraction window sizes (0 s corresponds to one sample). (**e**) Grand average time courses of the electrical potentials on electrode Cz with 95% confidence intervals.

We measured 10 participants with cervical SCI, each of whom was seated in a wheelchair in front of a computer screen. Instructions were given on the computer screen to the participants, and according to the instructions, they attempted or executed one of the following movements: pronation, supination, palmar grasp, lateral grasp or hand open. The offline paradigm is shown in Fig. 1. Participants executed or attempted a movement depending on their individual SCI status (see Table 1 and Supplementary Table 1)^37^. We performed an offline analysis and classified the 5 movements from the band-pass filtered EEG signals (0.3 to 3 Hz) with a shrinkage linear discriminant analysis (sLDA) classifier. This yielded a grand average accuracy, which peaked with 45.3% at 1.1 s after class cue presentation. The confidence interval at this peak was [40.3%, 50.3%] (calculated based on a t-distribution^38^). A plot of the participants’ classification accuracies and the grand average can be found in Fig. 2a. The plot includes the 95% confidence interval of the grand average (based on a t-distribution^38^). The chance level for 5 classes is 20% and the significance level for the grand average was determined as 22.3% (α = 0.05, adjusted wald interval^39,40^, Bonferroni corrected for the length of the analyzed time interval). Additionally, the peak accuracies for individual participants with their corresponding latencies are shown in Table 2. The confusion matrix in Fig. 2b, calculated at the peak of the grand average accuracy, shows that all classes are discriminable but classes involving common joints are more prone to be misclassified than classes involving separate joints. We classified EEG samples using a time window of 1.4 s length which was shifted along the trial, and aligned the classification accuracies to the center of this time window (we refer to this time window as feature extraction window). Similar to^31^, we then also compared different lengths of feature extraction windows. One can see in Fig. 2d and Supplementary Table 2 that the classification accuracies start saturating with feature extraction windows above 1 s. We therefore did not test feature extraction windows longer than 1.4 s and selected this window length for the classification analyses. The reader must keep in mind here that the filter and feature extraction windows were non-causal.

**Table 1:** Status of participants.

participant	sex	age [years]	dominant hand	tested hand	time since lesion [years | months]		AIS	NLI
P01	male	35	right	right	0	11	B	sub C6
P02	male	42	right	right	0	10	D	sub C1
P03	male	62	right	right	0	7	B	sub C5
P04	female	20	right	right	16	9	B	C5
P05	male	57	right	right	0	9	A	sub C4
P06	male	78	right	right	0	7	D	sub C5
P07	male	27	right	left	0	4	C	sub C4
P08	male	69	right	right	2	0	B	C7
P09	male	53	right	right	6	2	A	sub C4
P10	male	55	right	right	1	11	A	sub C6

Includes American Spinal Injury Association Impairment Scale (AIS) and Neurological Level of Injury (NLI). Explanation of AIS scores: A = complete, B = sensory incomplete, C = motor incomplete, D = motor incomplete.

**Table 2:** Peak classification accuracies per participant, and respective latencies with a feature extraction window size of 1.4 s.

participant	p01	p02	p03	p04	p05	p06	p07	p08	p09	p10
peak acc [%]	35.1	47.7	50.2	52.8	52.7	58.2	40.4	47.7	44.2	42.1
peak latency [s]	2.4	3.3	3.1	3.1	3	2.8	3.1	2.2	3.1	2.9

**Figure 3:**
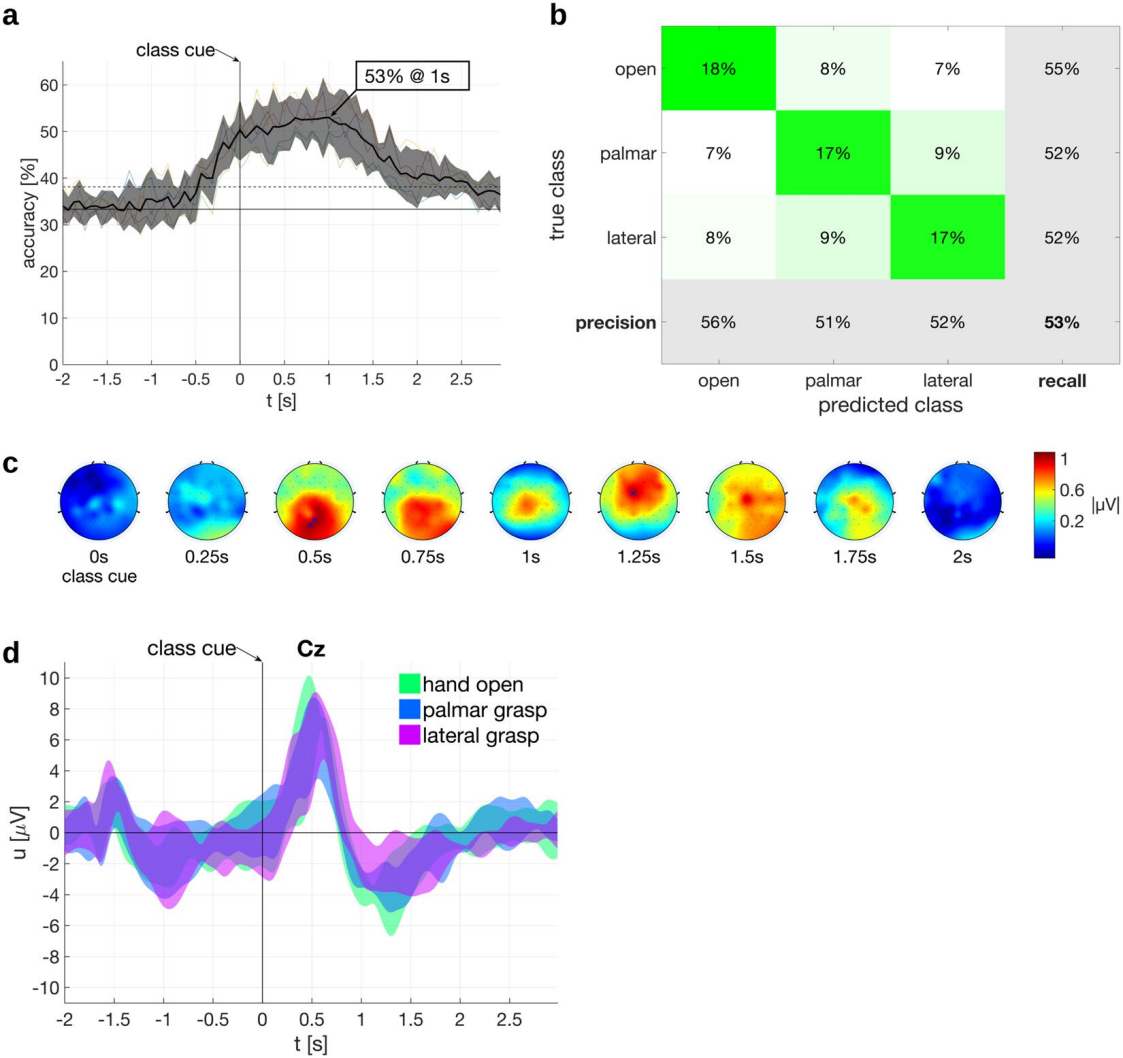
Offline Results for the subset of participants (n = 5) with no active hand movement comprising only of palmar grasp, lateral grasp and hand open classes. All times are relative to trial start (**a**) Classification accuracies of individual participants (thin traces) and the grand average (black thick line) with its respective 95% confidence interval. The classification accuracy peak is marked. (**b**) Confusion matrix at peak grand average classification accuracy. (**c**) Difference Topoplots. Statistically significant differences on electrodes are marked with an “x”. (**d**) Grand average time courses of the electrical potentials on electrode Cz with 95% confidence intervals.

The motor skills of our participants were not homogeneous and some of them had a remaining but impaired hand function or could rotate the forearm. We therefore selected a subset comprising 5 participants who have a motor score of 0 or 1 for finger flexors and finger abductors on the International Standards for Neurological Classification of Spinal Cord Injury (ISNCSCI) impairment scale^37^, i.e. no active hand movement. This subset was then subjected to a 3-class classification (palmar grasp, lateral grasp, hand open). By this means we analyzed exclusively those movement classes which could not be executed by the participants. Figure 3a shows the time course of the classification accuracies. The grand average peak was 53.0% at 1 s after class cue presentation with a 95% confidence interval of [47.5%, 58.6%] (the chance level is 33.3% and significance level is 38.1%). The confusion matrix in Fig. 3b indicates that all classes can be discriminated.

#### MRCPs

The trial averaged electrical potential on the central electrode Cz shows the typical pattern emerging in a synchronous paradigm (c.f. Fig. 2e). 500 ms after the presentation of the class cue, a positive peak developed corresponding to the cognitive processing of the cue. This positive peak is then followed by a negative peak around 1 s after cue presentation which we attribute to the movement attempt and related to MRCPs. Also the averaged potentials of the 5 participant subset (i.e. without active hand movement) have qualitatively similar features (c.f. Fig. 3d). Signals were non-causal filtered between 0.3 to 3 Hz with a monopolar reference. The 95% confidence intervals are based on a t-distribution^38^ over participants.

The early positive and the later negative peaks were defined as points-of-interest, and we analyzed whether their amplitude or latency contains discriminative information for the 5 movement classes. Descriptive statistics can be found in Table 3 which indicates differences of the peaks in the amplitudes and time latencies with respect to the movement classes. Moreover, a nonparametric Friedmann test (α = 0.05) found a statistically significant effect of the class on the amplitude of the positive and negative peak ([*χ*^2^(4) = 16.3, p = 0.0026], [*χ*^2^(4) = 20.3, p = 0.0004]), as well as on the latency of the positive and negative peak ([*χ*^2^(4) = 22.1, p = 0.0002], [*χ*^2^(4) = 12.2, p = 0.0158]). Thus, the early positive and the later negative peak contain discriminative information about the movement class.

**Table 3:** Descriptive statistics of the positive and negative peaks of the electrical potentials on Cz. Latency is relative to the class cue.

class	positive peak				negative peak			
	amplitude [µV]		latency [s]		amplitude [µV]		latency [s]	
	mean	std dev	mean	std dev	mean	std dev	mean	std dev
pronation	8.01	2.97	0.44	0.14	−5.48	2.38	1.10	0.09
supination	8.44	3.79	0.49	0.13	−6.05	2.15	1.05	0.11
hand open	8.17	3.36	0.51	0.11	−5.33	2.41	1.35	0.59
palmar grasp	6.56	3.34	0.53	0.08	−4.45	2.02	1.41	0.41
lateral grasp	6.68	2.86	0.56	0.08	−3.68	2.19	1.27	0.21

#### Difference topoplots

We calculated the differences between the topoplots of each class (see Section *Difference topoplots* for further explanations). This allowed us to check the origin of the brain signals containing discriminative information. The resulting plots are termed difference topoplots in this work, and can be seen in Figs 2c and 3c where we averaged over the full set of participants and the subset, respectively. For both sets, the difference topoplot sequences indicate differences on the central motor cortex, which peak around second 1. This means that at the time point of maximum classification accuracy, signals do indeed originate from plausible, i.e. movement-related, brain regions and not from other sources. The subset difference topoplots furthermore show an involvement of the occipital cortex around 1.5 s. Statistically significant differences at time points and electrodes are marked (α = 0.05, one-tailed nonparametric permutation test).

### Proof-of-Concept of an online classifier

We presented the offline classification of attempted movements in the previous section, here we introduce a proof-of-concept to demonstrate the classification of hand open vs palmar grasp in a closed-loop for a participant with SCI. We recruited participant P09 who has an ISNCSCI motor score of 0 for elbow extensors, finger flexors and finger abductors, i.e. total paralysis of the hand, and measured two sessions on different days.

**Figure 4:**
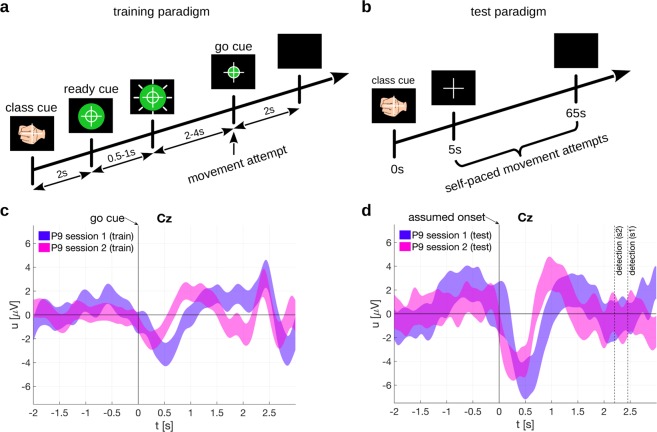
Training and test paradigms for online classification and respective electrode potentials. (**a**) Training paradigm. A green filled circle shrunk with a random speed. The participant attempted a movement (hand open or palmar grasp) when it hit the inner white circle, i.e. the go cue. (**b**) Test paradigm. The participant repeatedly attempted self-paced movements. (**c**) 95% confidence intervals of the electrode potentials on Cz, time locked to the go cue. (**d**) 95% confidence interval of the electrode potentials on Cz, time locked to the assumed movement onset (i.e. the movement detection time point corrected by the detection delay). Additionally, movement detection time points are shown for each session.

We introduced a new training paradigm for the classifier. One must keep in mind that the offline paradigm is synchronous and has a cue which requires a cognitive processing, which is then reflected in the EEG potential. Figure 2e is a convenient example of this. However, in a self-paced paradigm the cue-related potential would not be generated at all but only the MRCPs (associated to the negative peak). Therefore, it is imperative that the classifier is trained on MRCPs which are affected to the least extent possible by the cue. Figure 4a shows the training paradigm used for the proof-of-concept study. We used a training paradigm where the class cue was presented immediately at the trial start. This class cue was then replaced by the ready cue, a green filled circle, which started shrinking at a random speed. When the shrinking ball hit the circumference of a small white circle, representing the go cue, the participant was required to attempt the movement.

The trial-averaged electrode potential of Cz, time locked to the go cue (second 0), is shown in Fig. 4c. One can see in Fig. 4c that the positive peak before the negative peak is reduced. The depicted potentials include 95% confidence intervals based on a t-distribution^38^ over trials. The potentials corresponding to the two sessions differ right after the movement attempt at around 0.5 s to 1 s, which corresponds to the characteristic negative deflection of MRCPs. Interestingly, it was noted that there is a second negativity around 1.5 s to 2 s exclusively for the second session. This difference between sessions might be due to slightly different instructions to the participant. We asked for a sustained single movement attempt in the first session and for a short single movement attempt in the second session. The additional positive peak between 2 s and 2.5 s is well separated from the negative peak and could have been induced by the sudden disappearance of the cues on the computer screen at trial end. Overall, we can see that the potential time course is closer to that of typical MRCPs^25,26^. This training paradigm is therefore apparently more applicable to train a self-paced movement classifier than the offline paradigm. Using this new training paradigm and additional rest trials, we trained a 3-class online classifier to detect hand open, palmar grasp and rest state.

Subsequently, we evaluated the classifier using the test paradigm shown in Fig. 4b. The class cue (hand open, palmar grasp, rest) was shown together with a white cross in the beginning of a trial. After 5 s, the class cue disappeared and the white cross was shown for the next 60 s. The participant was instructed to perform in this 60 s period multiple self-paced movement attempts if it was a hand open or palmar grasp trial, or to remain inactive in rest trials. During recording, the online classifier was constantly active and provided feedback. When a movement was detected, a hand open or palmar grasp symbol was shown for 2 s on the computer screen. The participant was furthermore instructed to report a movement attempt by a soft speech sound 2 s after the movement attempt to mark movement events.

We defined then a true positive window relative to these reported events. We considered a movement detection only as a true positive when it occurred within the true positive window (regardless of the movement class). The true positive rate (TPR) is then the number of true positives (TP) divided by the number of reported movement attempts multiplied by 100. Formally, the TPR is defined as *TPR* = *TP/P*·100, where P is the number of condition positives (i.e. the sum of true positives and false negatives). The classification accuracy regarding hand open vs palmar grasp was calculated based on true positives only, i.e. detections outside the true positive window were ignored. We also calculate the number of false positives per minute (FP/min). To do this, every detection within a rest trial was counted as a false positive and then normalized to a per minute rate. Eventually, we obtained an average classification accuracy of 68.4% over both sessions, and the individual session results can be found in Table 4.

**Table 4:** Online detection and classification results of both sessions.

session	movement attempts	TP count	TPR [%]	FP/min	accuracy [%]	significance level [%]
1	188	50	26.6	3.2	66.0	62.4
2	130	48	36.9	3.6	70.8	65.3

**Figure 5:**
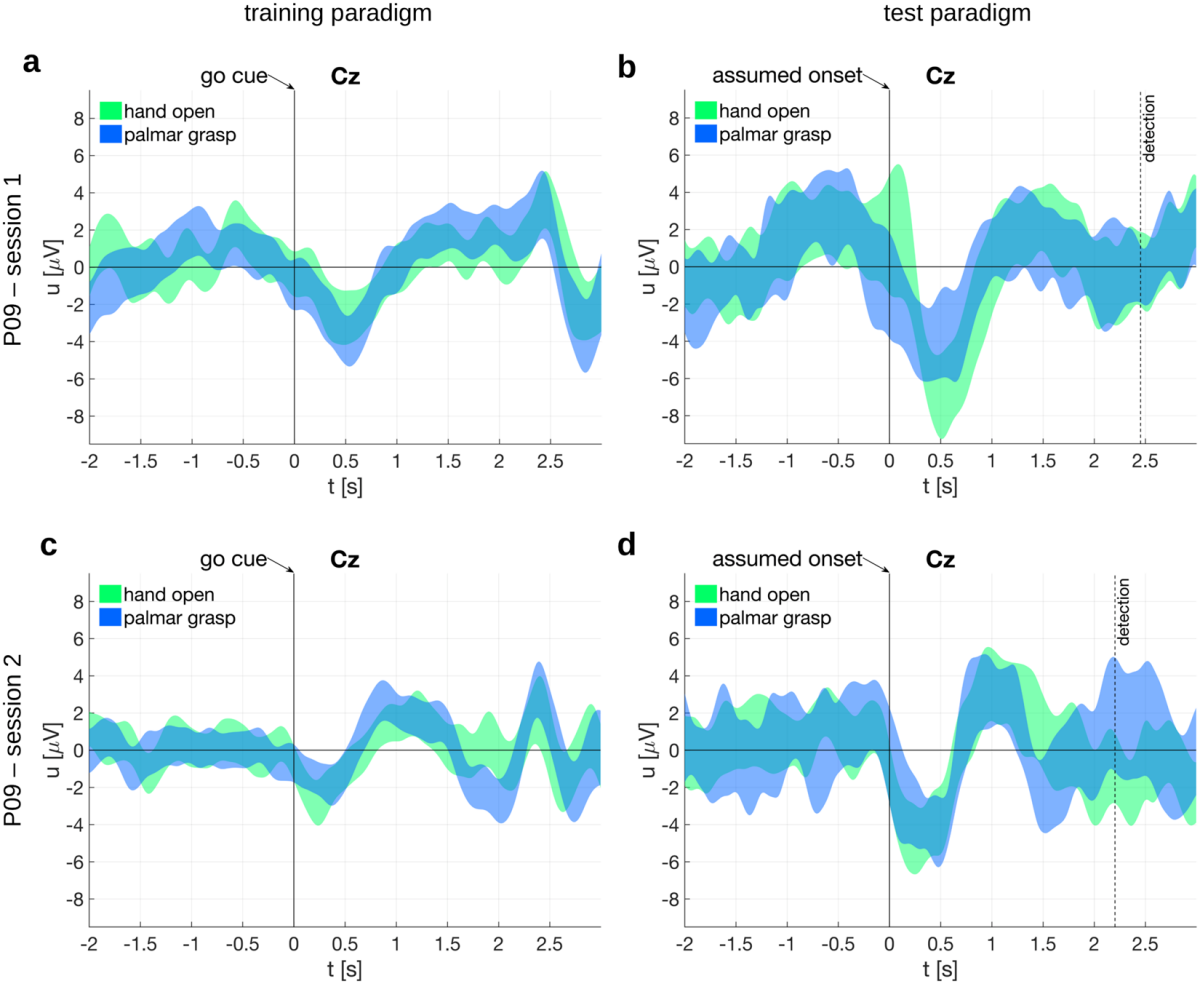
Electrode potentials at the proof-of-concept classification. Shown are the 95% confidence intervals at electrode Cz time locked to the go cue or assumed movement onset, respectively. (**a**) Session 1, training paradigm. (**b**) Session 1, test paradigm. (**c**) Session 2, training paradigm. (**d**) Session 2, test paradigm.

**Figure 6:**
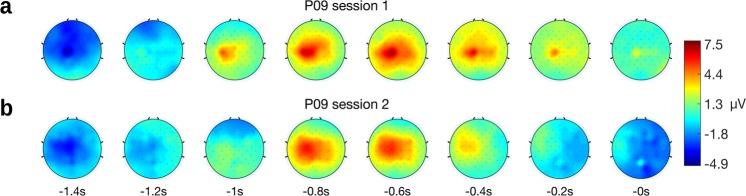
Topoplots from test paradigm. The topoplots are time-locked to true positive movement detections and second 0 corresponds to *t*_*train*_ (i.e. the detection time point minus 575 ms, see Section *Detection delay*). Thus, the time lags shown comprise the input features of the classifier. (**a**) Topoplot from session 1. (**b**) Topoplots from session 2.

Finally, we analyzed whether the classifier was indeed decoding from brain signals. First, we selected all true positives, i.e. all movement detections within the true positive window irrespective of the movement class. Next, we aligned to the assumed movement onset which is the time point of the movement detection minus the detection delay (c.f. Section *Detection delay*). The detection delay was 2.5 s in session 1 and 2.2 s in session 2. We then averaged over trials for each session, and show the electrode potential of Cz in Fig. 4d. The potentials show the typical negativity present in MRCPs and in the potentials elicited in the training paradigm, which is shown in Fig. 4c. The electrode potentials are shown separate for each movement class in Fig. 5. Second, we show in Fig. 6 the topoplots at the time lags used as features by the classifier (0 s to 1.4 s in 200 ms intervals). The topoplots (not to be confused with the difference topoplots) indicate that the classifier uses brain signals originating from lateral and central motor areas which is typical for MRCPs. Importantly, the EEG signals in Fig. 6 were causally filtered to show the reader what the classifier identified when it detected a true positive. The signals in Fig. 4c,d, however, were non-causally filtered to avoid phase distortion and ease the interpretation.

## Discussion

### Movement classification

We showed that participants with cervical SCI having impaired upper limb movements encode information about attempted arm and hand movements in low-frequency time-domain EEG signals. This information can be used to discriminate hand open, palmar grasp, lateral grasp, pronation, and supination.

We showed in our previous work the decoding of arm and hand movements from MRCPs^31,32^, i.e. low-frequency time domain signals. However, MRCPs are altered in participants with SCI^34–36^. It was therefore unclear if these participants encode similar information in the EEG as participants with an intact spinal cord. With respect to non-invasive recording techniques, it was so far only shown that low-frequency time-domain magnetoencephalography (MEG) signals can be used to discriminate a limited attempted movement set (hand open vs hand close) in participants with SCI^41^. To the best of our knowledge, this study is therefore the first to show that an extended arm/hand movement set can be classified from low-frequency time-domain EEG signals in participants with SCI.

We focused on low-frequency signals as they encode movement trajectories, movement directions, grasp types, speed or force in EEG^19–21,23,32,33,42–45^ and ECoG signals^46–51^. Other frequency bands used in classical oscillation-based BCIs^5,6,17^, i.e. mu and beta band, were found to encode a general movement state rather than movement parameters (how a movement is performed)^27,42,49,51–53^. However, recent studies extend these findings. For example, Iturrate *et al*.^33^ found spectral power differences in the upper alpha and lower beta bands for power and precision grasps. Korik *et al*.^54^ demonstrated the decoding of executed and imagined 3D hand movement trajectories from mu, beta, and low gamma bands. Future studies will have to further investigate the decoding of movement parameters from time-domain and frequency-domain features and consistently integrate the findings from both domains.

We found the EEG neural correlates of hand and arm movements differ in central brain areas. No apparent lateralization effect was observable. This is plausible as central brain areas like the Supplementary motor area (SMA), primary motor cortex (M1), and somatosensory cortex (S1) are involved in motor control^55,56^. However, a precise localization is not possible in the channel space. Furthermore, the accuracy plots and difference topoplots indicate that the movement information is spread over time. Moreover the electrical potential on Cz develops two distinctive peaks over time, a positive peak immediately after the cue followed by a negative peak. The positive peak is most likely a complex of P3 and positive slow wave potentials related to stimulus evaluation^57,58^ and response selection^59,60^; earlier perception related potentials are filtered out due to their higher frequency components. The negative peak can be explained as an MRCP^25,26^. Both peaks differ in their amplitude and latency depending on the movement, however only the negative peak around 3 s after cue leads to significant differences in the difference topoplots. Furthermore, we obtained significant classification accuracies at both peak latencies (especially with a short feature extraction window). We therefore make the assumption that processes related to inferring the desired behavior (evaluation of rules) as well as movement related processes can be decoded from low-frequency EEG signals.

We found as in^31,32^ that the classification accuracy improves with the length of the feature extraction window. This is expected as the discriminable information is spread over time. Moreover, the additional time inputs are weighted by the classifier which can be seen as a temporal filter. This filter could fine-tune the 0.3–3 Hz band-pass filter to extract the proper signals. The classification accuracies, however, show a saturation effect and time windows above 1 s add little or no improvement. Furthermore, we found that the discriminability is dependent on the joints involved in the movement. The confusion matrix indicates that movement classes with separate joints are better discriminable than movement classes with common joints, e.g. pronation is better discriminable from hand open than from supination. This was observed before in healthy participants^31^, and it implies that related body parts are encoded closer in low-frequency time-domain EEG signals than less related body parts. Thus, their signal sources are spatially closer and/or the time courses of the generated signals are more similar.

One can observe in the classification accuracy plots that the accuracy raises above significance level before the cue onset, i.e. at a time period when no class information was yet available to the participants. We ascribe that effect to the employed zero-phase band-pass filter and the symmetric feature extraction window which were non-causal. Due to these non-causal features and the low-frequency band, future information in the signal is perceptibly spread back in time. This leads to discriminable signals even before the class cue onset.

Participants with SCI available for studies are often heterogeneous with respect to their residual movement functions, and despite all participants having restrictions in their upper limb movements, the degree of movement function nevertheless varied from person to person. We therefore run an additional analysis comprised only of participants with no active hand movements and a restricted movement set (hand open, palmar grasp, lateral grasp). We obtained a statistically significant classification accuracy which peaked again around 1 s. Furthermore, the emerging neural correlates were similar. As before, differences in central brain areas are observable, but additionally also on occipital areas, which could be related to the visual processing of the cue. However, this effect vanished in a larger group and could be a spurious finding due to the small group size. Also the electrical potential on Cz has a qualitatively similar shape, comprising of a positive peak followed by a negative peak.

### Proof-of-concept of an online classifier

An online classifier ideally detects self-paced movements (i.e. movement vs. rest state), and simultaneously classifies them, for example as hand open or palmar grasp as in this work. The critical issue for an effective online classifier is the availability of applicable training data: the training paradigm should elicit MRCPs which are as close as possible to MRCPs elicited in a self-paced paradigm. However, cue-based paradigms similar to the Graz BCI paradigm^61^ are not suitable to elicit clean MRCPs because they are usually contaminated with cue-related potentials (as we have observed in the first part of this study). An alternative would be a self-paced training paradigm where the classifier is trained by time-locking on the movement onset^62–67^. However, participants with SCI may have lost all movement functionality of the respective limb, and may not provide any measurable EMG signals to detect the movement onset. We therefore designed a suitable training paradigm and added ready and go cues in addition to the class cue. The class cue requires the retrieval and selection of memorized task rules, and separates these processes from the actual movement attempt. There were variable time intervals before the go cue in order to make class and ready cue associated potentials indiscernible when time-locking to the go cue and averaging. Another important point in our paradigm is that the ready and go cues have a class-independent appearance to avoid any class-related perception differences. Plus, by adding a fixation cross in the middle and a symmetrically shrinking green ball, eye movements can be minimized. Finally, both cross and green ball are still displayed for 2 seconds after the go cue to avoid further evoked potentials close to the go cue due to their disappearance. Furthermore, the predictability of the go cue as given by our training paradigm is critical. The literature shows that MRCPs preceding self-paced and regular-cued movements are similar in shape and topography^68–70^. On the downside, our training paradigm may be contaminated with a contingent negative variation (CNV) potential, which is elicited by a warning stimulus followed by an imperative stimulus^71,72^. The CNV comprises of two components, one occurs after the warning stimulus and one before the imperative stimulus (i.e. the go cue in our paradigm). The last component in particular could form a potential complex with the Bereitschaftspotential (BP)^26^. However, for longer intervals between the warning and imperative stimulus (in the second range), the second component of the CNV and the late BP become similar in time course and scalp distribution^73–75^. Aside from these theoretical considerations, the potentials measured on participant P09 resemble the shape of band-pass filtered MRCPs. Interestingly, around 1.5 s to 2 s after the go cue we can observe a sustained positive potential deflection in training session 1 which is absent in training session 2. This difference in the potentials is probably due to differences in the movement strategy. Participant P09 was instructed to sustain the attempted movement until the end of the trial in training session 1 but not in training session 2. However, the majority of the EEG samples used for classifier training are from an interval before the deflection differences occur. The effect of the movement strategy on the evaluation outcome should be therefore negligible, which is also indicated by comparable classification accuracies in the two test sessions.

Interestingly, the hand open vs palmar grasp vs rest classifier had its maximum offline classification accuracy at *t*_*train*_ = 1.875 s (session 1) and *t*_*train*_ = 1.625 s (session 2), respectively. This means that the classifier found - using a 1.4 s long feature extraction window - the EEG samples from 0.475 s to 1.875 s (session 1), and from 0.225 s to 1.625 (session 2) most discriminative. Thus, the classifier was trained on an interval which started just before the negative potential peak on Cz and also covered the following positive deflection. This differs from the classifier in the offline paradigm, which on average used samples symmetrically around the negative peak. However, this shift could have been caused by the fact that we used a non-causal filter with the offline paradigm and/or that no rest class was present.

In addition to the training paradigm, we also designed an online test paradigm. This test paradigm elicited MRCPs which are similar in morphology to the MRCPs found in the training paradigm: they show a notable negative deflection followed by a positive deflection on Cz. This is to be expected since a classifier detects the trained pattern. However, the test MRCPs have larger amplitudes than the training MRCPs. One reason for this could be that self-paced movements cause similar yet more pronounced potential peaks than predictably cued movements^70^. Another reason could be the detection logic based on pre and post classes. It could be that the strict detection thresholds – which had to be exceeded by the pre, post and movement class – only allowed the detection of very pronounced MRCPs. Furthermore, the associated topoplots are interesting as they show a broad but central negativity followed by a lateralized positivity. This indicates that the classifier has indeed decoded brain signals, i.e. MRCPs, and not movement induced artefacts. However, the test paradigm was designed for a first proof-of-concept and this took place in a controlled environment, to avoid external influences. Future studies will need to evaluate and improve the classifier’s generalization capabilities as MRCPs depend on various factors, like force and speed^30,76^, goal-directedness^77^, attention diversion^78^, or externally or internally selected movement types^79^. This generalization is essential in the process of translating the research to the practical needs of end-users.

We obtained a TPR of around 30%, with more than 3 FP/min for participant P09. TPR and FP were calculated regardless of the movement class (hand open or palmar grasp). Furthermore, we obtained a classification accuracy of 68.4%. The classification accuracy was calculated using true positives only. False positives do not coincide with an intention to move and would impede the interpretation of the classification accuracy. The separation of TPR and FP/min from the classification accuracy allows evaluation of the movement detection performance separately from the movement classification. The detection of movements from MRCPs, particularly lower limb movements, is well reported in the literature^30,44,62–65,67,80^. Lower-limb MRCPs-based movement detection performances up to a TPR of 82.5% with FP/min of 1.38 are reported^62^, which are nearly 3 times better than the performance reached in this work. However, those results were obtained in healthy participants, and lower limb movements produce more pronounced MRCPs than upper limb movements^81^. What is novel in our work is the simultaneous online classification of various self-paced hand movement attempts in a participant with SCI using EEG. It is noteworthy that we tested our classification approach on a participant with a chronic and complete cervical SCI, and without any remaining hand function (AIS A, NLI C4). Thus, an intact spinal cord is not a prerequisite to classify movements from MRCPs. To the best of our knowledge, only a study based on MEG applied a related protocol for movement online classification in participants with SCI^41^, although this was cue-based. In this MEG study, two out of five paralyzed participants reached a significant and comparable classification accuracy of 83.3% and 66.7%, respectively, on classifying hand open vs hand close movement attempts.

The participant group size in the offline analysis (n = 10) is in the range of typical BCI studies but rather small compared to typical medical or general neuroscience standards. While we backed up all findings with state-of-the-art analysis and statistics, spurious findings cannot be fully excluded. Nevertheless, the classification and imaging findings are consistent with the literature, which strengthens our confidence in the interpretation of the results. Because of the limited group size and the resulting localization error, we refrained to do an analysis of the difference topoplots in the source space or to discuss the origin of the signal sources in detail. Moreover, we would like to point out that the band-pass filter was not systematically optimized with respect to the classification performance but based on previous studies^31,32^. Furthermore, the online classifier is a proof-of-concept. Other classification approaches based on, e.g. recurrent neural networks or hidden markov models for time series may provide a better detection and classification performance. In addition, we would like to highlight that the online training paradigm was in fact a cue-based paradigm. The influence of the class cue has been diminished, but a true self-paced training paradigm could yield more authentic MRCPs and therefore a better classification and detection performance. Moreover, we demonstrated our online classifier with a single person with SCI, and therefore cannot make any predictions of the expected performance or its variance in the population of persons with SCI.

We showed that various movement attempts of the upper limb can be classified offline from low-frequency time-domain EEG signals in participants with SCI. Furthermore, we have introduced a proof-of-concept on how to detect and classify movements in quasi real-time in a closed-loop setup. While the performance of the classifier is probably not yet sufficient to be used for neuroprosthesis control, we were able to show for the first time the general feasibility of classifying different single upper limb movements in an end-user with SCI.

## Methods

### Movement classification

#### Participants

We recruited and measured 10 participants with subacute and chronic cervical SCI in a rehabilitation center (AUVA rehabilitation clinic, Tobelbad, Austria). They were aged between 20 and 69 years and suffered their lesion 3 months to 16 years before the study. Inclusion criteria were legal age of 18 years and restricted hand function, whereas artificial ventilation was an exclusion criterion. Their neurological level of injury (NLI) ranged from C1 to C7 and their American Spinal Injury Association Impairment Scale (AIS) score ranged from A to D. See Table 1 and Supplementary Table 1 for details. Written informed consent was obtained from all participants or a witness. The study was conducted in accordance with the protocol approved by the ethics committee for the hospitals of the Austrian general accident insurance institution AUVA (approval number 3/2017).

#### Paradigm

Each of the participants sat in front of a computer screen with an arm resting on a pillow on their lap or on a table and they carried out the instructions given on the computer screen. At the trial start, a fixation cross and a beep sound were presented. We asked the participants to focus their gaze firmly on the cross which was displayed during the whole trial period of 5 s to avoid eye movements, see Fig. 1a. Furthermore, we instructed participants to avoid swallowing and eye blinking during the trial period. The class cue was displayed 2 s after the trial start for 3 s (i.e. until the end of the trial) and corresponded to one of 5 classes: pronation, supination, palmar grasp, lateral grasp or hand open (c.f. Fig. 1b). Based on the participants’ residual motor abilities (c.f. Supplementary Table 1), they were asked to execute or attempt the corresponding movement immediately when the class cue appeared. Furthermore they were asked to avoid any other movement during the current cue phase. If the participants were able to execute a movement, they went back to their initial rest position after the trial period. Between trials, a break with a random period of 1 s to 3 s followed. We recorded 9 runs with 40 trials per run, i.e. 72 trials per class in total.

#### Recording

We measured the EEG with 61 electrodes covering frontal, central, parietal and temporal areas. Additionally, we measured the electrooculogram (EOG) with 3 electrodes placed above the nasion and below the outer canthi of the eyes. Reference was placed on the left earlobe and ground on AFF2h. Signals were recorded using four 16-channel g.USBamps biosignal amplifiers and a g.GAMMAsys/g.LADYbird active electrode system (g.tec medical engineering GmbH, Austria) with 256 Hz and a band-pass filter from 0.01 Hz to 100 Hz (8^th^ order Chebyshev filter). Power line interference was suppressed with a notch filter at 50 Hz.

#### Preprocessing

Signals were processed using Matlab R2017a (MathWorks, Massachusetts, USA) and the external toolboxes BioSig 3.3.0^82^ and EEGLAB 14.1.1b^83,84^. In short, first (1) we removed noisy channels, next (2) we removed stationary artefacts with ICA, we then (3) detected trials with transient artefacts, and finally (4) we removed stationary and transient artefacts from a narrow band EEG signal (0.3–3 Hz). It is noteworthy that we performed steps 2 and 3 on the EEG signal using a broader frequency range of 0.3–70 Hz to ease the detection of artefacts. The ICA weights were then cached and subsequently applied in step 4.

First (1), we visually inspected signals and removed channels contaminated with perceptible noise. Additionally, we removed by default channel AFz as it is sensitive to eye blinks and eye movements.

Next (2), we removed stationary artefacts by filtering the signals from 0.3 Hz to 70 Hz (4^th^ order Butterworth zero-phase band-pass filter) and computed independent components (ICs) with the extended infomax independent component analysis (ICA) implemented in EEGLAB. We reduced the data dimensionality before computing the ICA with a principal component analysis (PCA) and retained principal components explaining 99% of the variance of the data. Furthermore, we computed the ICA only on samples with an absolute value of less than 10.4 times the median absolute deviation (MAD)^85^ of a channel. MAD is robust deviation measure and the chosen threshold corresponds to 7 times the standard deviation for normally distributed data. We then identified the ICs contaminated with muscle and eye related artefacts, removed those ICs and projected the remaining ICs back to the original space.

Next (3), we detected transient artefacts using EEGLAB and marked trials for rejection with (1) values above/below −100 μV and 100 μV, respectively, (2) trials with abnormal joint probabilities, and (3) trials with abnormal kurtosis. The methods (2) and (3) used as threshold 5 times the standard deviation of their statistic to detect artefact contaminated trials.

Finally (4), we removed the previously computed and artefact contaminated ICs from the original data (i.e. 0.01–100 Hz filtered). For this purpose we applied the cached ICA weights, removed previously identified artefactual components, and back-projected to the channel space. Lastly, we applied a zero-phase 4^th^ order Butterworth band-pass filter from 0.3 Hz to 3 Hz as in^31^, and excluded trials marked for rejection from further processing.

#### Classification

We re-referenced the preprocessed EEG signals to a common average reference (CAR) and classified the EEG with a multiclass shrinkage linear discriminant analysis (sLDA) classifier^86,87^. The input to the sLDA classifier were the EEG samples from all low noise channels. Furthermore, we used multiple causal and non-causal time points of the EEG as an input to the classifier. For this purpose, EEG samples spaced in 200 ms intervals were taken from a time window, i.e. the feature extraction window, and fed into the classifier (here we tested 8 different window lengths from 0 s to 1.4 s, see Supplementary Table 3 for the feature number). The output of the classifier was normalized with a softmax function to obtain probabilities.

We shifted the feature extraction window along the trial in steps of 1/16^th^ of a second, and calculated classification accuracies aligned to the center of the feature extraction window. Classification accuracies were validated with a trial-based 10 × 10 cross-fold validation.

#### Difference topoplots

The EEG channel layout corresponds to the 10-5 system^88^. Noisy channels which were excluded in the preprocessing step were interpolated with a biharmonic spline interpolation (griddata command in Matlab). We calculated the differences of the trial averaged electrode potentials for all class combinations (i.e. palmar grasp vs lateral grasp, hand open vs lateral grasp, etc.). Next, we computed the absolute values of all differences as we were only interested in the intensity of the differences and not in any polarity, and averaged over all class combinations. Finally, we averaged over non-overlapping 250 s time segments from 0 s to 3 s relative to the class cue. We refer to these average absolute potential differences as difference topoplots.

We found significant differences on channels and time points with a one-tailed nonparametric permutation test^89,90^ with α = 0.05. For this purpose, we shuffled class labels once for each participant and computed shuffled difference topoplots. The test statistic was the difference between the shuffled and unshuffled difference topoplots. We accounted for multiple comparisons by calculating the permutation distribution using the maximum test statistic over channels and time points.

### Proof-of-concept of an online classifier

#### Paradigm

We employed two separate paradigms in the online classification, one to train the classifier (training paradigm), and one to evaluate the performance (test paradigm). The training paradigm comprises two different trial types: movement trials and rest trials. In a movement trial (c.f. Fig. 4a), a class cue together with a cross and a beep were displayed in the beginning of a trial. The class cue represented either hand open or palmar grasp. After 2 s, the class cue was replaced by the ready cue, a filled green circle with a smaller inner white circle. After a random time interval of 0.5 s to 1 s, the filled green circle started to shrink with a random speed to the size of the inner white circle in 2 s to 4 s. The participant was instructed to attempt the movement corresponding to the class cue when the filled green circle hit the inner white circle, i.e. the go cue. In session 1, we instructed the participant to attempt to open or grasp, and deliberately hold the position until the end of the trial, i.e. attempt a sustained movement. In session 2, we gave the instruction not to hold the position, but to make a short single movement attempt. In both sessions, the experimenter demonstrated the participant a hand open and a palmar grasp movement executed at a regular speed, and asked the participant to attempt to imitate these movements. The screen was then cleared 2 s later at the end of the trial. A break of 2 s to 3 s was between trials. The other trial type was a rest trial, where a cross was shown for 70 s and the participant was instructed to avoid any movement during this period. We recorded 5 movement runs, each comprises 30 movement trials, and 4 rest runs each comprises 1 rest trial. Thus, in total we recorded 150 movement trials (75 trials per movement class) and 4 rest trials.

In the test paradigm, the class cue (hand open, palmar grasp, rest), a fixation cross, and a beep were presented at the beginning of a trial. The class cue was then removed at 5 seconds and a 60 s long period of movement or rest, followed. In the case of a rest class cue, we instructed the participant to avoid any movement during this period. In the case of a movement related class cue, we instructed the participant to attempt multiple self-paced movements of the requested movement class during the 60 s period, see Fig. 4b. Furthermore, we instructed the participant to report any movement attempt 2 s afterwards by a soft speech sound. The experimenter then promptly pressed a button on the computer to mark the time point of a movement event. However, due to a misunderstanding, the participant reported movement attempts immediately afterwards in session 1, which is also reflected in different true positive window offsets (see Section *Definition of the true-positive window*). Moreover the participant was instructed to wait at least 3 s after reporting before attempting the next movement. The online classifier was constantly active and showed the corresponding movement icon (hand open or palmar grasp) for 2 s whenever a movement attempt was detected. We then recorded 6 runs in session 1 and 5 runs in session 2. Each run comprised of 4 movement trials and 1 rest trial.

#### Online classifier and detection thresholds

Recording, preprocessing, and classification were performed in a manner similar to the descriptions in Section *Movement classification* except that the feature extraction window and band-pass filter were causal and no ICA artifact removal was applied. Using the training paradigm (see Fig. 4a) and the previously described classifier with a 1.4 s long feature extraction window, we classified 3 classes: hand open, palmar grasp and rest. Rest trials were obtained by epoching the 70 s long original rest trials into 150 trials. Thus, the number of rest trials was equal to the total number of movement trials. We then calculated the offline classification accuracies on the time interval 1 s to 2 s after the go cue with a 10-fold cross-validation, and found the time point *t*_*train*_ with the highest offline classification accuracy (*t*_*train*_ = 1.875 s in training session 1, *t*_*train*_ = 1.625 s in training session 2, respectively). In addition to the 3 original movement/rest classes, we introduced a pre class and a post class to increase the robustness of the classifier. The pre and post classes respectively detect the early and late phases of MRCPs. These phases could otherwise increase the chance of detecting a wrong class if the MRCPs, which are spread over time, are not yet (or are no longer) fully covered by the feature extraction window. The pre class and post class features were gathered for this purpose from *t*_*train*_ − 500 ms and *t*_*train*_ + 500 ms, respectively. That is, the feature extraction window was shifted by −500 ms and 500 ms relative to *t*_*train*_. Pre class and post class comprised features from both hand open and palmar grasp trials but no rest trials. The final online classifier was then trained with the classes hand open, palmar grasp, rest, pre and post. If the participant attempted a movement, an ideal output of the online classifier would show a peak of the pre class probability, followed 500 ms later by a peak of the hand open or palmar grasp class probability, and another 500 ms later by a peak of the post class probability. See Supplementary Fig. 1c,d for examples of the trial averaged classifier output.

Finally, we defined 3 time windows to detect the pre, movement, and post class probability peaks. Each time window was specified by time length and time position relative to a reference position *t*_0_. The pre window ranged from −650 ms to −350 ms relative to *t*_0_; the movement window ranged from −50 ms to 50 ms; the post window ranged from 350 ms to 650 ms. A movement was detected when all the following conditions were met: (1) pre class probability is above 0.7 for at least 150 ms within the pre window, (2) hand open or palmar grasp class probability is above 0.9 during the movement window, and (3) post class probability is above 0.7 for at least 150 ms within the post window. The movement class (hand open or palmar grasp) with the higher probability within the movement window was then eventually detected. After a detection, a refractory period of 2 s was imposed. Supplementary Fig. 2 illustrates the thresholds and time windows. The thresholds were set before the evaluation and were not subsequently changed.

#### Detection delay

Different causes led to a delay between the movement attempt and the detection of the movement attempt. We assume that the participant started the movement attempt exactly at the go cue in the training paradigm. First, due to the time extension of the MRCPs, the filter delay, and the 1.4 s long feature extraction window, the point with the highest classification accuracy is delayed from the go cue and is given by *t*_*train*_. Second, we introduced a delay by our pre, movement and post window based detection logic. We provide in the following a conservative estimation of the expected detection delay, i.e. the average maximum delay, under the assumption that the average post class classifier output is maximal 500 ms after *t*_*train*_ and symmetric (c.f. Supplementary Fig. 1c,d). This is a reasonable assumption, since the post class is trained on data 500 ms after *t*_*train*_. For a conservative estimation, we assume that the average post class classifier output crosses the probability-threshold (0.7) for exactly the length of the time-threshold (150 ms, see the description of the detection logic in the previous section). Shorter probability-threshold crossings do not cause a detection, and longer crossings cause an earlier detection. The total average maximum detection delay then comprises (1) *t*_*train*_, (2) the time delay from *t*_*train*_ to the center of the post window (500 ms), and (3) half of the time-threshold (150/2 ms; the other half is already covered by the previous point). Thus, the maximum detection delay between the movement attempt and the detection time point is on average *t*_*train*_ + 500 ms + 75 ms.

It is a conservative estimation because crossings of the post window probability-threshold for longer than 150 ms – but still centered on *t*_*train*_ + 500 ms – cause earlier crossings of the 150 ms time-threshold, and therefore shorter movement detection delays, see Supplementary Fig. 2. An exact assessment of the average detection delay would require the knowledge of the exact pre, movement, and post class probability distributions in the test paradigm which we cannot measure. Furthermore, we estimated the average of the detection delay and not the single trial detection delays which are not possible to determine.

#### Definition of the true-positive window

In order to evaluate the performance of the online classifier, we defined a true positive window and counted every detection within it as a true positive, and every detection outside it as a false positive. We set the length of the true-positive window to 2 s which allowed for a maximum of one detection due to the refractory period. The center of the true-positive window was set by an offset relative to the time points of the reported movement attempts, whereby the true-positive window should capture the assumed movement onset (i.e. the movement detection corrected by the detection delay). Thus, the offset should correspond to the average time difference between the assumed movement onset and the movement event marked by the experimenter. As the offset is not known a priori (i.e. there is no ground truth regarding the start of the movement attempt), we employed a systematic approach to determine it. We iterated the offset from 0 s to 5 s and calculated for each offset value the TP/FP ratio, to which we refer as detection ratio. The offset which maximized the detection ratio was then used as the offset for a session (leading to an offset of 2.2 s in session 1 and 4.2 s in session 2). See Supplementary Fig. 1a,b for the dependency of the detection ratio and classification accuracy on the offset.

As a remark, the FPs in the movement trials were solely used to determine the offset of the true-positive window but not to determine the FP/min rate shown in the results section. The FP/min rate was determined exclusively from rest trials.

## Supplementary information

Supplementary Information

## Data Availability

Data are available from the BNCI Horizon 2020 database at http://bnci-horizon-2020.eu/database/data-sets (accession number 001–2019) and from Zenodo at 10.5281/zenodo.2222268.
