# Supplementary material for: Attempted Arm and Hand Movements can be Decoded from Low-Frequency EEG from Persons with Spinal Cord Injury
Source: Sci Rep. 2019 May 9;9:7134. doi: 10.1038/s41598-019-43594-9 (PMC6509331; doi:10.1038/s41598-019-43594-9)
Supplement: Supplementary file 1 — Supplementary Information [file 41598_2019_43594_MOESM1_ESM.pdf]

Supplementary Information

Attempted Arm and Hand Movements can be Decoded from Low-Frequency EEG from Persons with Spinal Cord Injury

Patrick Ofner[1], Andreas Schwarz[1], Joana Pereira[1], Daniela Wyss[2], Renate Wildburger[2], Gernot R. Müller-Putz[1]

[1]Graz University of Technology, Institute of Neural Engineering, BCI-Lab, Graz, Austria
[2]AUVA rehabilitation clinic, Tobelbad, Austria

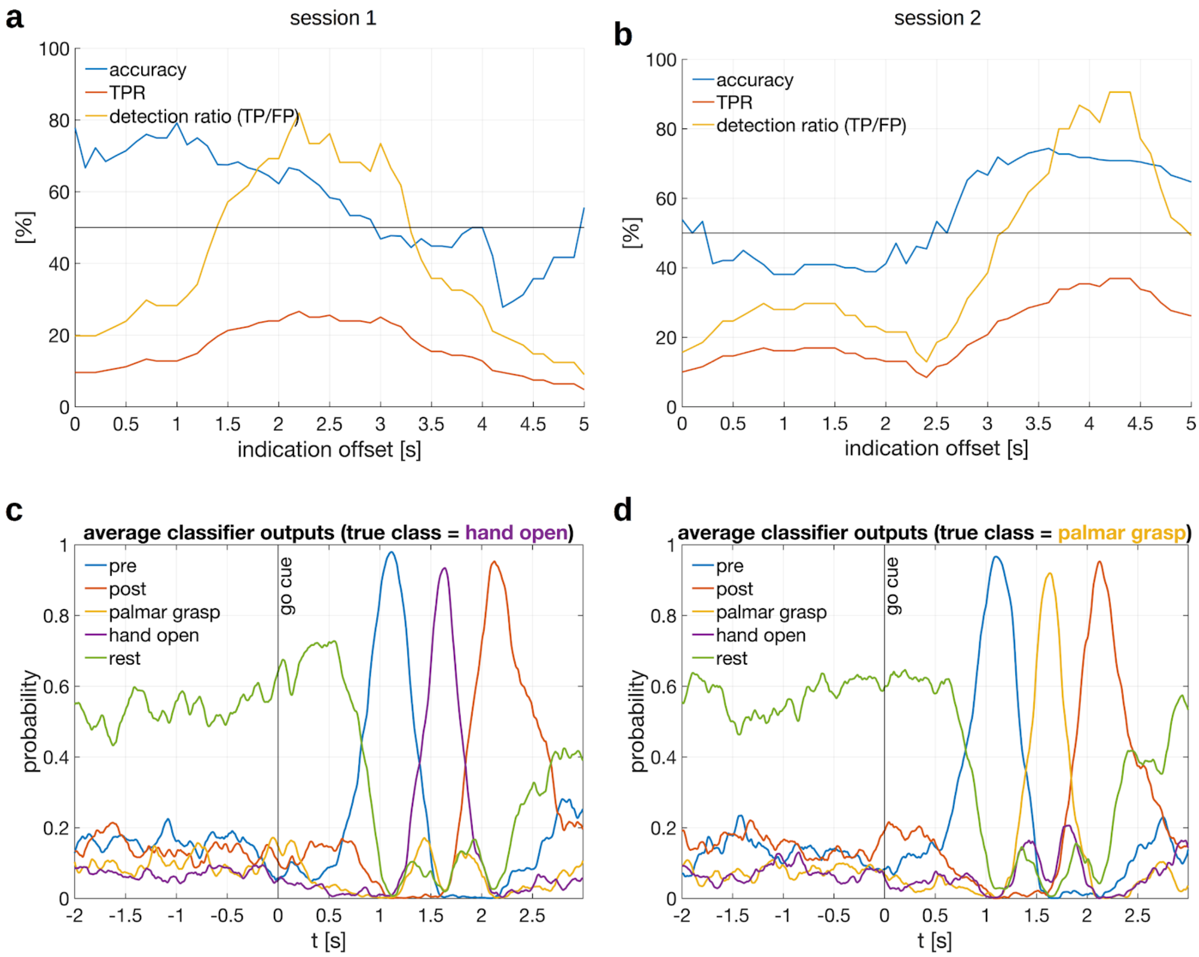

Supplementary Figure 1: **a** & **b**: Accuracy, true positive rate (TPR) and detection ratio (TP/FP) in relation to the reporting offset for session 1 and 2 for participant P09 in the test paradigm. **c**: Trial averaged classifier output of hand open trials in the training paradigm. **d**: Trial averaged classifier output of palmar grasp trials in the training paradigm.

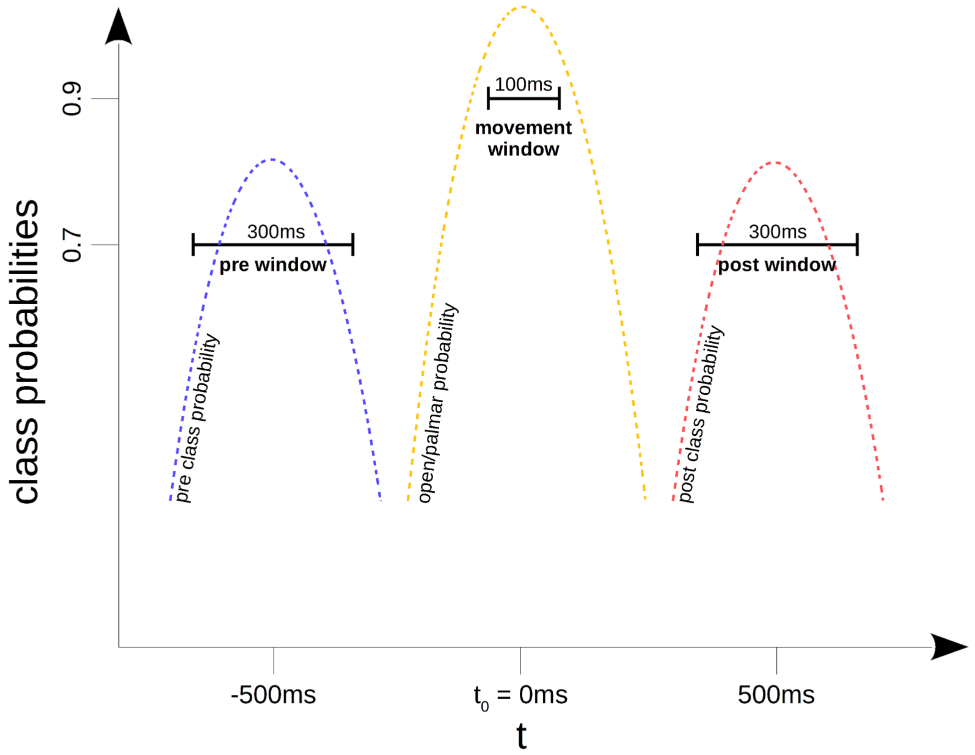

Supplementary Figure 2: Illustration of the pre, movement and post windows, and how they are used to detect class probability peaks. A movement attempt was detected when the pre, open/palmar and post class probabilities peaked in sequential order: the pre class probability had to be higher than 0.7 for at least 150ms within the pre window (and analogous for the post class); the hand open or palmar grasp probability had to be higher than 0.9 during the whole movement window.

Supplementary Table 1: **ISNCSCI motor scores of tested limb.**

motor key muscles		*P01*	*P02*	*P03*	*P04*	*P05*	*P06*	*P07*	*P08*	*P09*	*P10*
elbow flexors	C5	5	3	4	5	0	4	4	5	4	4
wrist extensors	C6	5	3	4	4	0	4	5	5	1	4
elbow extensors	C7	2	3	3	2	0	4	4	4	0	4
finger flexors	C8	1	3	1	0	0	4	4	2	0	0
finger abductors	T1	0	1	1	0	0	NT	3	2	0	0

Supplementary Table 2: **Comparison of the grand average classification accuracy peaks for different feature extraction window sizes (from 0 - 1.4s).**

window size	0s	0.2s	0.4s	0.6s	0.8s	1.0s	1.2s	1.4s
peak acc avg. [%]	33	38	41.3	43.3	45.2	45.3	45.1	45.3
peak acc std. dev. [%]	5.5	5.3	5.9	5.5	5.9	6.3	6.9	1
peak latency [s]	2.9	2.6	2.6	2.8	2.8	2.8	2.9	3.1

Supplementary Table 3: **Number of features in dependence on feature extraction window length.** Figures assume that all channels but AFz are included.

window length [s]	0	0.2	0.4	0.6	0.8	1.0	1.2	1.4
features/channel	1	2	3	4	5	6	7	8
features	60	120	180	240	300	360	420	480
